# Sociodemographic Characteristics and Comorbidities of Patients With Long COVID and Persistent Olfactory Dysfunction

**DOI:** 10.1001/jamanetworkopen.2022.30637

**Published:** 2022-09-08

**Authors:** Alna Carolina Mendes Paranhos, Ápio Ricardo Nazareth Dias, Livia Caroline Machado da Silva, Gisele Vieira Hennemann Koury, Emanuel de Jesus Sousa, Antônio José Cerasi, Givago Silva Souza, Juarez Antônio Simões Quaresma, Luiz Fábio Magno Falcão

**Affiliations:** 1 Tropical Medicine Center, Federal University of Pará, Belém, Brazil; 2 Biological and Health Center, Pará State University, Belém, Brazil; 3 Biological Science Center, Federal University of Pará, Belém, Brazil; 4 Cosmopolita College, Belém, Brazil; 5 University of São Paulo, São Paulo, Brazil

## Abstract

**Importance:**

Determining the characteristics, type, and severity of olfactory dysfunction in patients with long COVID is important for the prognosis and potential treatment of the affected population.

**Objective:**

To describe the sociodemographic and clinical features of patients with long COVID who develop persistent olfactory dysfunction.

**Design, Setting, and Participants:**

This cross-sectional study, conducted at a rehabilitation center at a public university in the Amazon region of Brazil between September 9, 2020, and October 20, 2021, comprised 219 patients with long COVID and self-reported neurologic symptoms. Of these 219 patients, 139 received a diagnosis of chronic olfactory dysfunction, as confirmed by the Connecticut Chemosensory Clinical Research Center (CCCRC) test.

**Exposure:**

Clinical diagnosis of long COVID.

**Main Outcomes and Measures:**

Electronic case report forms were prepared for the collection of sociodemographic and clinical data. Patients’ sense of smell was evaluated via a CCCRC test, and the association of olfactory dysfunction with aspects of daily life was recorded using a questionnaire.

**Results:**

Of the 219 patients included in the study, 164 (74.9%) were women, 194 (88.6%) were between 18 and 59 years of age (mean [SD] age, 43.2 [12.9] years), 206 (94.1%) had more than 9 years of education, and 115 (52.5%) had a monthly income of up to US $192.00. In the study group, 139 patients (63.5%) had some degree of olfactory dysfunction, whereas 80 patients (36.5%) had normosmia. Patients with olfactory dysfunction had a significantly longer duration of long COVID symptoms than those in the normosmia group (mean [SD], 242.7 [101.9] vs 221.0 [97.5] days; *P* = .01). Among patients with anosmia, there was a significant association between olfactory dysfunction and daily activities, especially in terms of impairment in hazard detection (21 of 31 patients [67.7%]), personal hygiene (21 of 31 patients [67.7%]), and food intake (21 of 31 patients [67.7%]). Univariable logistic regression analyses found that ageusia symptoms were associated with the occurrence of olfactory dysfunction (odds ratio [OR], 11.14 [95% CI, 4.76-26.07]; *P* < .001), whereas headache (OR, 0.41 [95% CI, 0.22-0.76]; *P* < .001) and sleep disorders (OR, 0.48 [95% CI, 0.26-0.92]; *P* = .02) showed an inverse association with the occurrence of olfactory dysfunction.

**Conclusions and Relevance:**

Olfactory dysfunction is one of the most important long-term neurologic symptoms of COVID-19, with the highest prevalence seen among women, adults, and outpatients. Patients with olfactory dysfunction may experience persistent severe hyposmia or anosmia more than 1 year from the onset of symptoms, suggesting the possibility of the condition becoming a permanent sequela.

## Key Points

**Question:**

What are the sociodemographic and clinical characteristics of patients with long COVID and persistent olfactory dysfunction?

**Findings:**

In this cross-sectional study of 219 patients with long COVID and neurologic symptoms, 64% had olfactory dysfunction, with the highest prevalence among women, adults, and outpatients. Patients with olfactory dysfunction may develop severe olfactory loss (hyposmia or anosmia) that may persist for more than 1 year after the onset of symptoms.

**Meaning:**

This study suggests that olfactory dysfunction in patients with long COVID may become permanent.

## Teaser

This cross-sectional study describes the sociodemographic and clinical features of patients with long COVID who develop persistent olfactory dysfunction.

## Introduction

Long COVID can be described as a set of symptoms, signs, or abnormal laboratory test parameters persisting for 2 weeks or more after the onset of COVID-19.^1,2,3,4^ In total, 55 long-term effects of COVID-19 have been identified, with fatigue, lung dysfunction, abnormal chest radiograph results, neurologic disorders, and anosmia being the most common.^5^ Olfactory dysfunction is among the most prevalent neurologic symptoms among patients with long COVID, and persistent anosmia has been reported in 23% of patients with acute COVID-19.^5,6,7^

To date, little is known regarding the long-term course of olfactory dysfunction associated with COVID-19, and the question of whether it is completely reversible remains unclear.^8,9^ However, chronic olfactory disorders are associated with disturbances in eating behavior, depression, and a general reduction in quality of life.^10,11,12^ Individuals with chronic olfactory dysfunction report difficulties with cooking, maintaining health and nutritional status, personal hygiene, and social relationships^10,13^ and are 3 times more likely to experience hazardous events, such as smoke, delayed detection of gas leaks, and spoiled food.^14^

The characteristics, type, and severity of olfactory dysfunction are important in determining prognosis and potential treatment.^15,16^ This study aimed to describe the sociodemographic and clinical features of patients with long COVID who developed persistent olfactory dysfunction and its features and the association of persistent olfactory dysfunction with daily life activities.

## Methods

### Study Population

This cross-sectional study was conducted among individuals enrolled in a follow-up program for long COVID at a public university in Belém, in the Amazon region of Brazil. The genetic heterogeneity and admixture of the Amazon population are characteristics that overlap the concepts of isolated races; therefore, race and ethnicity were not discussed in this study. Patients had long COVID and a history of typical symptoms of acute COVID-19 with positive nasal swab reverse transcription–polymerase chain reaction results and presented with long-duration symptoms.^2^ None of the study participants had been immunized prior to SARS-CoV-2 infection. This study was approved by the Ethics Committee on Research with Human Beings of the State University of Pará and followed the ethical principles of the Declaration of Helsinki.^17^ All participants provided written informed consent. This observational, cross-sectional, quantitative, descriptive, and analytical study followed the Strengthening the Reporting of Observational Studies in Epidemiology (STROBE) reporting guideline^18^ for cross-sectional studies and the Standards of Reporting of Neurological Disorders (STROND) checklist—a guideline for reporting of incidence and prevalence in neuroepidemiology studies.^19^

Participants were registered using an electronic form (Google Forms; Google Corp) and were later contacted via telephone for a formal neurologic consultation with a multidisciplinary team. Patients (n = 250) older than 18 years were evaluated between September 9, 2020, and October 20, 2021. Of these 250 patients, 31 were excluded because of a previous history of head trauma, neurologic disease, or neurosurgery, resulting in a sample size of 219 patients. Among the 219 patients with confirmed long-term neurologic symptoms, 139 presented with chronic olfactory dysfunction associated with SARS-CoV-2 infection (Figure).

**Figure.**
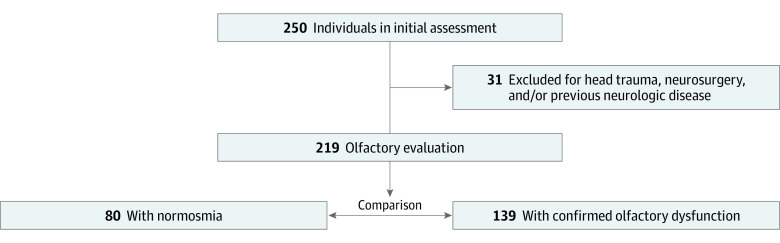
Flowchart of the Study

### Study Design, Data Collection, and Procedures

We collected sociodemographic and clinical data, including variables such as educational level, sex, income, health history (diabetes, allergic rhinitis, and smoking), reported symptoms (headache, depression, anxiety, insomnia, tingling in extremities, mild cognitive disorder, tiredness, dyspnea, weakness, and pain), anosmia, and ageusia after COVID-19 from an electronic medical record (Microsoft Access; Microsoft Corp).

We evaluated patients’ sense of smell with the Connecticut Chemosensory Clinical Research Center (CCCRC) test. The CCCRC test solutions were developed by the pharmacology laboratory of Cosmopolita College, Belém, Brazil, following the recommendations of Cain et al^20^ and Fenólio et al.^21^ The CCCRC test is composed of 2 subtests: a threshold test and an odor identification test.

For the threshold test, a test kit comprising 8 bottles was used; 7 bottles contained different dilutions of butanol (n-butyl alcohol) in distilled water at the following butanol concentrations: 0.005%, 0.01%, 0.05%, 0.1%, 0.4%, 1%, and 4%; and 1 bottle contained only distilled water. The procedure consisted of 2 alternatives with a forced choice between the butanol concentration and distilled water. The concentration of the first solution with 4 correct identifications was considered the perceptual threshold for the identification of the smell of butanol. The odor identification test consisted of identification through inhalation of 8 common substances to be identified from a 16-item list.

The CCCRC score for each nostril is calculated by adding the score of the threshold and odor identification tests. The score can range between 0 and 7. The final score was arrived by taking the mean of the scores of both nostrils. The final score classified the participants as having anosmia (0-1.75), severe hyposmia (2-3.75), moderate hyposmia (4-4.75), mild hyposmia (5-5.75), and normosmia (6-7) (eFigure in the Supplement).

In addition, we evaluated the association of olfactory dysfunction with daily life activities using a questionnaire that listed domains in which olfactory function plays a major role according to previous studies: (1) In your opinion, did the olfactory dysfunction affect your personal hygiene? Yes or no? (personal hygiene: ability to keep the body clean and sensing of body odors); (2) In your opinion, did the olfactory dysfunction affect your food intake? Yes or no? (food intake: ability and behavior related to eating); (3) In your opinion, did the olfactory dysfunction affect your preparation of food ability? Yes or no? (preparation of food: ability and behavior related to cooking); (4) In your opinion, did the olfactory dysfunction affect your hazard detection ability? Yes or no? (hazard detection: ability to detect environmental hazards [eg, spoiled food, gas leak, fire, or smoke]); (5) In your opinion, did the olfactory dysfunction affect your work? Yes or no? (work: activities related to the development, production, delivery, or management of objects or services); and (6) In your opinion, did the olfactory dysfunction affect your social relations? Yes or no? (social relations: activities that involve social interaction with others, including family, friends, peers, and community members). The questionnaire was self-completed, and patients were instructed to mark only domains directly affected by COVID-19–associated olfactory dysfunction.

### Statistical Analysis

Descriptive results are presented as mean (SD) values for continuous variables and frequencies and percentages for categorical variables. Data normality was assessed using the D’Agostino-Pearson *K*^2^ test. Parametric variables were assessed with the 2-sample *t* test, and nonparametric variables were assessed with the Mann-Whitney test or the χ^2^ test. Univariable and multivariable logistic models estimated the odds ratios (ORs) that were used to evaluate the association between olfactory dysfunction and the independent variables (female sex, age, no hospital admittance, headache, sleep disorder, depression, anxiety, ageusia, tingling of extremities, mild cognitive disorder, fatigue, and time from onset symptoms) of the sample. All *P* values were from 2-sided tests, adjusted for multiple comparisons, and results were deemed statistically significant at *P* ≤ .05. Statistical analysis was performed using GraphPad Prism, version 5.0 software (GraphPad Software).

## Results

Of the 219 patients in the study, 164 (74.9%) were women, 194 (88.6%) were between 18 and 59 years of age (mean [SD] age, 43.2 [12.9] years), 206 (94.1%) had more than 9 years of education, and 115 (52.5%) had a monthly income of up to US $192 (Table 1). A total of 80 patients (36.5%) had normosmia, and 139 (63.5%) had some degree of olfactory dysfunction.

**Table 1.** Comparison of Sociodemographic and Clinical Features Among Study Population According to the CCCRC Classification

Feature	Patients, No. (%)			*P* value^a^
	General (N = 219)	Normosmia (n = 80)	Olfactory dysfunction (n = 139)	
Age, y				
18-59	194 (88.6)	67 (83.8)	127 (91.4)	.12
≥60	25 (11.4)	13 (16.3)	12 (8.6)	
Sex				
Female	164 (74.9)	62 (77.5)	102 (73.4)	.49
Male	55 (25.1)	18 (22.5)	37 (26.6)	
Years of education				
≤9	13 (5.9)	7 (8.8)	6 (4.3)	.23
>9	206 (94.1)	73 (91.3)	133 (95.7)	
Monthly income, US$				
≤192.00	115 (52.5)	39 (48.8)	76 (54.7)	.39
>192.00	104 (47.5)	41 (51.3)	63 (45.3)	

Abbreviation: CCCRC, olfactory test of Connecticut Chemosensory Clinical Research Center.

^a^ Considering normosmia × olfactory dysfunction groups.

Table 2 provides the clinical characteristics of the participants with olfactory dysfunction grouped according to the CCCRC test score, as well as the comparison between the normosmia and olfactory dysfunction groups. There was no significant difference in the hospitalization rates between the normosmia group and the olfactory dysfunction groups (16 of 80 [20.0%] vs 19 of 139 [13.7%]; *P* = .21). The olfactory dysfunction group had a significantly longer duration from symptom onset than the normosmia group (mean [SD], 242.7 [101.9] vs 221.0 [97.5] days; *P* = .01), and the olfactory dysfunction group had a higher proportion of participants with neurologic symptoms for more than 6 months than the normosmia group (110 of 139 [79.1%] vs 51 of 80 [63.8%]; *P* = .01). More patients with normosmia than patients with olfactory dysfunction had headache (43 of 80 [53.8%] vs 52 of 139 [37.4%]; *P* = .01), sleep disorder (29 of 80 [36.3%] vs 32 of 139 [23.0%]; *P* = .03), and anxiety (36 of 80 [45.0%] vs 30 of 139 [21.6%]; *P* < .001) symptoms, whereas the olfactory dysfunction group had a higher proportion of patients with ageusia (83 of 139 [59.7%] vs 19 of 80 [23.8%]; *P* < .001).

**Table 2.** Clinical Findings Among Study Population According to CCCRC Classification

Characteristic	Patients, No. (%)		*P* value	Olfactory dysfunction, No. (%) of patients			
	Normosmia (n = 80)	Olfactory dysfunction (n = 139)		Mild hyposmia (n = 21)	Moderate hyposmia (n = 23)	Severe hyposmia (n = 64)	Anosmia (n = 31)
Hospitalization							
Yes	16 (20.0)	19 (13.7)	.21	5 (23.8)	4 (17.4)	4 (6.3)	6 (19.4)
No	64 (80.0)	120 (86.3)		16 (76.2)	19 (82.6)	60 (93.8)	25 (80.6)
Time from symptom onset, mean (SD), d	221.0 (97.5)	242.7 (101.9)	.01^a^	264.4 (119.4)	246.2 (105.6)	243.9 (101.7)	222.7 (87.2)
Time from symptom onset, mo							
≤6	29 (36.3)	29 (20.9)	.01^b^	3 (14.3)	5 (21.7)	14 (21.9)	7 (22.6)
>6	51 (63.8)	110 (79.1)		18 (85.7)	18 (78.3)	50 (78.1)	24 (77.4)
Health history							
Allergic rhinitis	12 (15.0)	33 (23.7)	.12	4 (19.0)	6 (26.1)	14 (21.9)	9 (29.0)
Smoking	3 (3.8)	5 (3.6)	.95	0	0	2 (3.1)	3 (9.7)
Diabetes	8 (10.0)	9 (6.5)	.34	2 (9.5)	0	6 (9.4)	1 (3.2)
Long COVID symptoms							
Ageusia	19 (23.8)	83 (59.7)	<.001^b^	12 (57.1)	12 (52.2)	39 (60.9)	21 (67.7)
Fatigue	31 (38.8)	67 (48.2)	.17	15 (71.4)	9 (39.1)	33 (51.6)	10 (32.3)
Cognitive disorder	40 (50.0)	62 (44.6)	.44	13 (61.9)	9 (39.1)	27 (42.2)	13 (41.9)
Headache	43 (53.8)	52 (37.4)	.01^b^	13 (61.9)	11 (47.8)	20 (31.3)	8 (25.8)
Weakness	14 (17.5)	31 (22.3)	.39	10 (47.6)	4 (17.4)	13 (20.3)	4 (12.9)
Dyspnea	14 (17.5)	27 (19.4)	.72	6 (28.6)	4 (17.4)	9 (14.1)	8 (25.8)
Paresthesias	16 (20.0)	35 (25.2)	.38	8 (38.1)	3 (13.0)	18 (28.1)	6 (19.4)
Sleep disorder	29 (36.3)	32 (23.0)	.03^b^	8 (38.1)	5 (21.7)	14 (21.9)	5 (16.1)
Anxiety	36 (45.0)	30 (21.6)	<.001^b^	8 (38.1)	5 (21.7)	13 (20.3)	4 (12.9)
Depression	11 (13.8)	17 (12.2)	.74	5 (23.8)	5 (21.7)	6 (9.4)	1 (3.2)

Abbreviation: CCCRC, olfactory test of Connecticut Chemosensory Clinical Research Center.

^a^ Calculated using a 2-sample *t* test with a threshold for statistical significance of *P* ≤ .05.

^b^ Calculated using a χ^2^ test with a threshold for statistical significance of *P* ≤ .05.

Hazard detection, personal hygiene, and food preparation were the domains of daily life most frequently associated with olfactory dysfunction. The anosmia group reported impairments more frequently than the other olfactory dysfunction subgroups, with significant differences in personal hygiene (anosmia, 21 of 31 [67.7%]; mild hyposmia, 7 of 21 [33.3%]; moderate hyposmia, 13 of 23 [56.5%]; severe hyposmia, 29 of 64 [45.3%]), food intake (anosmia, 21 of 31 [67.7%]; mild hyposmia, 7 of 21 [33.3%]; moderate hyposmia, 9 of 23 [39.1%]; severe hyposmia, 24 of 64 [37.5%]), and hazard detection (anosmia, 21 of 31 [67.7%]; mild hyposmia, 7 of 21 [33.3%]; moderate hyposmia, 14 of 23 [60.9%]; severe hyposmia, 31 of 64 [48.4%]) (Table 3).

**Table 3.** Long-term Association of Olfactory Dysfunction With Domains of Daily Life

Domain	Olfactory dysfunction (n = 139)	Hyposmia, No. (%) of patients			Anosmia (n = 31)	*P* value^a^
		Mild (n = 21)	Moderate (n = 23)	Severe (n = 64)		
Personal hygiene	70 (50.4)	7 (33.3)	13 (56.5)	29 (45.3)	21 (67.7)	.05^b^
Food intake	61 (43.9)	7 (33.3)	9 (39.1)	24 (37.5)	21 (67.7)	.02^b^
Preparation of food	65 (46.8)	9 (42.9)	11 (47.8)	24 (37.5)	21 (67.7)	.16
Hazard detection	73 (52.5)	7 (33.3)	14 (60.9)	31 (48.4)	21 (67.7)	.05^b^
Work	28 (20.1)	4 (19.0)	4 (17.4)	12 (18.8)	8 (25.8)	.58
Social relations	48 (34.5)	6 (28.6)	7 (30.4)	22 (34.4)	13 (41.9)	.21
Not answer	19 (13.7)	4 (19.0)	2 (8.7)	11 (17.2)	2 (6.5)	NA

Abbreviation: NA, not applicable.

^a^ Adjusted for multiple comparisons between mild hyposmia, moderate hyposmia, severe hyposmia, and anosmia.

^b^ Calculated using a χ^2^ test with a threshold for statistical significance of *P* ≤ .05.

On univariable logistic regression for epidemiologic and clinical characteristics, olfactory dysfunction was associated with ageusia (OR, 11.14 [95% CI, 4.76-26.07]; *P* < .001) and inversely associated with headache (OR, 0.41 [95% CI, 0.22-0.76]; *P* < .001) and sleep disorders (OR, 0.48 [95% CI, 0.26-0.92]; *P* = .02) (Table 4). On multivariable logistic regression, olfactory dysfunction was significantly associated with ageusia symptoms (OR, 13.24 [95% CI, 5.24-33.47]; *P* < .001).

**Table 4.** Association of Olfactory Dysfunction and Severe Olfactory Dysfunction With Clinical Features of the Study Population (N = 219)

Clinical feature	Olfactory dysfunction (CCCRC score <6)				Severe olfactory dysfunction (CCCRC score <3)			
	Univariable analysis		Multivariable analysis		Univariable analysis		Multivariable analysis	
	OR (95% CI)	*P* value	OR (95% CI)	*P* value	OR (95% CI)	*P* value	OR (95% CI)	*P* value
Female	0.62 (0.30-1.31)	.21	0.42 (0.17-1.05)	.06	1.20 (0.65-2.24)	.56	1.22 (0.61-2.45)	.57
Age (18-59 y)	1.89 (0.86-4.19)	.11	1.89 (0.71-5.05)	.19	1.72 (0.71-4.19)	.22	1.26 (0.47-3.41)	.64
No hospital admission	2.05 (0.97-4.38)	.06	1.14 (0.46-2.85)	.76	2.14 (0.98-4.72)	.05	1.34 (0.55-3.30)	.51
Headache	0.41 (0.22-0.76)	<.001	0.56 (0.27-1.22)	.14	0.35 (0.20-0.63)	<.001	0.36 (0.19-0.71)	.002
Sleep disorder	0.48 (0.26-0.92)	.02	0.64 (0.28-1.50)	.31	0.48 (0.26-0.91)	.02	0.50 (0.23-1.14)	.09
Depression (BDI score, >20)	0.58 (0.31-1.13)	.10	0.95 (0.38-2.36)	.91	0.63 (0.36-1.14)	.13	0.80 (0.35-1.87)	.61
Anxiety (BAI score, >20)	0.65 (0.36-1.21)	.17	0.71 (0.31-1.67)	.43	0.82 (0.48-1.41)	.48	1.04 (0.49-2.22)	.91
Ageusia	11.14 (4.76-26.07)	<.001	13.24 (5.24-33.47)	<.001	3.19 (1.83-5.58)	<.001	2.82 (1.53-5.21)	<.001
Tingling extremities	0.96 (0.48-1.95)	.92	1.63 (0.65-4.12)	.29	1.21 (0.65-2.28)	.54	2.23 (1.00-5.00)	.05
Mild cognitive disorder	0.72 (0.40-1.31)	.28	0.85 (0.38-1.90)	.69	0.72 (0.42-1.25)	.24	0.95 (0.49-1.85)	.88
Tiredness or fatigue	1.03 (0.57-1.89)	.90	1.47 (0.66-3.29)	.34	1.03 (0.61-1.78)	.89	1.41 (0.72-2.78)	.30
Dyspnea	0.86 (0.41-1.84)	.70	0.49 (0.19-1.31)	.15	0.9 (0.46-1.81)	.78	0.78 (0.35-1.77)	.56
Time from onset of symptoms (>6 mo)	1.64 (0.86-3.16)	.13	2.11 (0.95-4.68)	.06	1.58 (0.86-2.90)	.13	1.76 (0.90-3.45)	.09

Abbreviations: BAI, Beck Anxiety Inventory; BDI, Beck Depression Inventory; CCCRC, olfactory test of Connecticut Chemosensory Clinical Research Center; OR, odds ratio.

On univariable analysis for the epidemiologic and clinical characteristics associated with severe olfactory dysfunction (CCCRC score, <3), inverse associations were found for headache (OR, 0.35 [95% CI, 0.20-0.63]; *P* < .001) and sleep disorders (OR, 0.48 [95% CI, 0.26-0.91]; *P* = .02); no hospitalization (OR, 2.14 [95% CI, 0.98-4.72]; *P* = .05) demonstrated a significant association with severe olfactory dysfunction (Table 4). Tingling was associated with severe olfactory dysfunction in multivariable analysis (OR, 2.23 [95% CI, 1.00-5.00]; *P* = .05), and ageusia was associated with severe olfactory dysfunction in both analyses (univariable: OR, 3.19 [95% CI, 1.83-5.58]; *P* < .001; multivariable: OR, 2.82 [95% CI, 1.53-5.21]; *P* < .001).

## Discussion

Loss of smell was the most reported neurologic symptom among the 219 patients in this study with long COVID, primarily seen among women, adults, and those not hospitalized during the acute phase of COVID-19. Patients with olfactory dysfunction after COVID-19 may develop severe degrees of olfactory loss (severe hyposmia or anosmia) even 1 year after the onset of symptoms, suggesting the possibility of permanent sequelae. The daily life activities most associated with olfactory dysfunction among patients with anosmia were personal hygiene, food intake, and prevention of accidents. Logistic regression analyses found that ageusia was the only risk factor associated with the occurrence of olfactory dysfunction, whereas headache and sleep disorders showed an inverse association with the occurrence of olfactory dysfunction.

Previous studies found similar results, and olfactory dysfunction was associated with female sex,^22,23,24,25^ middle age,^22,24,25^ outpatient clinical course,^23,24,25,26,27^ and lower probability of being admitted to the hospital owing to COVID-19.^26,27^ One hypothesis is that a small, focused viral load of SARS-CoV-2 in the upper airways may lead to mild infection and consequently decreases the risk of overloading the host’s immune response and hospitalization.^15,25^

Most COVID-19–associated olfactory dysfunctions are transient, lasting approximately 2 to 3 weeks.^22,27^ This finding is consistent with the fact that SARS-CoV-2 has a high affinity for the sustentacular cells of the olfactory epithelium that express angiotensin-converting enzyme 2 (ACE2) and possess substantial capacity for repair and regeneration after damage.^28,29,30,31,32^ However, epithelial injury secondary to ACE2-mediated entry does not completely explain the inverse association between olfactory dysfunction and disease severity.

A recent report proposed a second route for viral entry mediated by neuropilin 1 (NRP1), an immune cell expressed by regulatory T cells that exerts immunosuppressive effects.^33^ Neuropilin 1 is abundant in all olfactory cells and can facilitate direct damage to olfactory receptor neurons and, consequently, the olfactory bulb. The authors argued that variability in NRP1 expression by age, race and ethnicity, or sex may explain the differing levels of morbidity of infection and the inverse association between anosmia and COVID-19 severity. A higher expression of NRP1 may lead to a higher risk of olfactory dysfunction but greater activation of regulatory T cells that suppress a cytokine storm.^33^ Our results support this hypothesis.

Conversely, the absence of ACE2 expression by olfactory sensory neurons has weakened the neurotropic potential of patients with COVID-19, suggesting that olfactory dysfunction is not associated with viral damage to neuronal cells and other areas of the central nervous system.^29,34^ The pathologic changes in the central nervous system may originate from the hematogenous route and spread through the blood-brain barrier.^35,36^ These different mechanisms of viral entry may explain the inverse association found between the occurrence of olfactory dysfunction and other central nervous system disorders, such as headache and sleep disorders.

The mild forms of COVID-19 in the acute phase contrast with the persistence and severity of olfactory dysfunction among patients with long COVID.^37,38,39^ Possible causes of prolonged olfactory dysfunction after COVID-19 include damage to basal cells, continuous inflammation, and chronic SARS-CoV-2 infection in the olfactory epithelium.^29^ Chronic inflammation could modulate gene expression and switch the function of olfactory epithelium basal cells from neural regeneration to inflammatory signaling and immune cell proliferation.^40^

In our study, we found a long-term association of olfactory dysfunction with all domains of daily life listed in our questionnaire, especially among patients with anosmia. It is known that patients with chronic olfactory dysfunction have considerable disruption to their daily life^37,41,42^ and have the highest risk of developing mood disorders, such as anxiety and depression,^43,44^ and neurodegenerative diseases, such as Parkinson disease and Alzheimer disease.^45,46,47^

Several studies have used psychological tests to evaluate olfactory dysfunction among patients with COVID-19. The use of objective tests to evaluate alterations in smell is strongly encouraged when compared with subjective assessments based on the patients’ perception.^48,49^ The Sniffin’ Sticks test is the most commonly used test.^16,49,50^ Other olfactory sensitivity tests include the University of Pennsylvania Smell Identification Test,^51^ the Toyota & Takagi Olfactometer,^52^ the Cross-Cultural Smell Identification Test,^53^ the Brief Smell Identification Test,^54^ and the CCCRC test.^20^ In the present investigation, the entire cohort was evaluated using the CCCRC test, which is used worldwide and has the advantages of low cost and the possibility of large-scale clinical use.^55^ In addition, a recent version of the CCCRC test has been validated in the Brazilian population.^21^

To our knowledge, this is the first study conducted in a Brazilian population containing a large number of patients with long COVID, attributing internal validity to our results. These data are important because the prevalence rates of olfactory dysfunction among patients with COVID-19 appear to vary between populations; for example, a recent report found that White individuals are 3 times more likely to develop olfactory dysfunction (54%) than Asian individuals (17.7%).^55,56,57^

The prevalence of olfactory dysfunction was high (63.5%) in our study; a possible explanation would be the predominance of wild-type SARS-CoV-2 infection in our study population because almost all of the participants reported the onset of symptoms around March, April, and May 2020. A recent study showed that anosmia associated with COVID-19 is more frequent and severe among patients infected with the wild-type virus than among those infected with the Delta variant (B.1.167.2), as well as increasing the likelihood of chronic olfactory dysfunction.^58^

High recovery rates of persistent olfactory dysfunctions are expected within 1 year^8^; however, despite our analyses occurring within this period, the group with olfactory dysfunction had long COVID for a significantly longer time than the group with normosmia, and most patients with olfactory dysfunction had severe dysfunction. Long periods of severe olfactory dysfunction are associated with worse diagnosis and risk of permanent sequelae.^59,60,61^ Other studies have found similar results with a high prevalence of olfactory dysfunction after 6 months^39,62^ and reinforce the finding that a marked proportion of patients do not recover quickly.

### Limitations

This study has some limitations. Dysgeusia was not tested with psychophysical tests, which could mean 2 confounding factors in our analyses: (1) an overestimation of this symptom in our sample, because retronasal smell and true dysgeusia are often confused by patients, and (2) it is not known whether the reported difficulties in food intake, also assessed subjectively, are a consequence of the severity of the olfactory dysfunction, dysgeusia, or both. Because long COVID is characterized by a series of overlapping symptoms, it is possible that some associations with the quality-of-life domains are the result of interactions between the symptoms and not just the olfactory dysfunction in isolation.

In this study, qualitative olfactory disorders, such as parosmia, phantosmia, and cacosmia, were not analyzed. The data are being collected as part of the follow-up research of this cohort and will be published in the future because the presence of these olfactory dysfunctions may be associated with the recovery of sense of smell.^63,64^

The absence of formal data regarding previous clinical history and the acute phase of COVID-19 is a potential confounding factor, which was minimized by carefully using an anamnesis form and specialized consultation with neurologists and otolaryngologists. Future studies should continue the monitoring of this population, prioritizing interdisciplinary research in clinical, epidemiologic, and basic science, such as genetics and immunology. These data should not be generalized because they were from a single center in 1 region of Brazil. The data, however, serve as a benchmark for further studies.

## Conclusions

The results of our investigation reaffirmed that olfactory dysfunction is one of the most important long-term neurologic symptoms of COVID-19, with the highest prevalence among women, adults, and outpatients. We observed in this cohort that patients with olfactory dysfunction may experience persistent severe hyposmia or anosmia more than 1 year from the onset of symptoms, suggesting the possibility of permanent sequelae.

Our results highlight the need to continue monitoring the rate of recovery of olfactory function among individuals with long COVID to evaluate whether it is a chronic or permanent sequela. In addition, clinical trials and longitudinal studies are recommended to verify the effectiveness of potential treatments and the postulated risk for an increase in neurologic sequelae or neurodegenerative disorders in this population.

## Notes

Supplement. **eFigure.** Description of Connecticut Chemosensory Clinical Research Center Test Procedures and Score Calculation
