# Supplementary material for: Sociodemographic Characteristics and Comorbidities of Patients With Long COVID and Persistent Olfactory Dysfunction
Source: JAMA Netw Open. 2022 Sep 8;5(9):e2230637. doi: 10.1001/jamanetworkopen.2022.30637 (PMC9459661; doi:10.1001/jamanetworkopen.2022.30637)
Supplement: Supplement. — eFigure. Description of Connecticut Chemosensory Clinical Research Center Test Procedures and Score Calculation [file jamanetwopen-e2230637-s001.pdf]

Supplementary Online Content

Mendes Paranhos AC, Nazareth Dias ÁR, Machado da Silva LC, et al. Sociodemographic characteristics and comorbidities of patients with long COVID and persistent olfactory dysfunction. *JAMA Netw Open.* 2022;5(9):e2230637. doi:10.1001/jamanetworkopen.2022.30637

eFigure. Description of Connecticut Chemosensory Clinical Research Center Test Procedures and Score Calculation

This supplementary material has been provided by the authors to give readers additional information about their work.

© 2022 Mendes Paranhos AC et al. *JAMA Network Open.*

eFigure. Description of Connecticut Chemosensory Clinical Research Center Test Procedures and Score Calculation

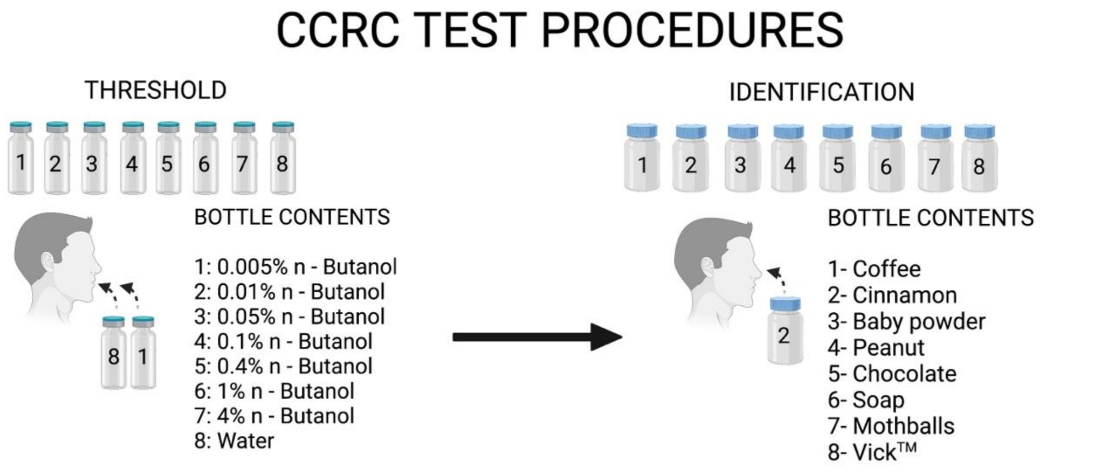

SCORE CALCULATION

	Right Nostril	Left Nostril	Combined (Left + Right)
Threshold	x	y	x + y ÷ 2
Odor Identification	w	z	w + z ÷ 2
Score of each Nostril	x + w ÷ 2	y+z ÷ 2	(x + y ÷ 2) + (w + z ÷ 2) ÷ 2<br>**Final Score**

Final Score	Classification
0 – 1.75	Anosmia
2 – 3.75	Severe Hyposmia
4 – 4.75	Moderate Hyposmia
5 – 5.75	Mild Hyposmia
6 - 7	Normosmia

Legends: To determine the olfactory threshold, individuals smell different concentrations of n-butanol (as listed). The score is from 0 to 7, where minor concentration corresponds to major score (7), and error (non-detection of n-butanol) corresponds to 0. To determine the identification of odours, individuals smell different substances (as listed). The score corresponds to the number of assertions in a list of different substances. The final score and classification are determined as showed in table.

© 2022 Mendes Paranhos AC et al. *JAMA Network Open.*
